# CLAP: A web-server for automatic classification of proteins with special reference to multi-domain proteins

**DOI:** 10.1186/1471-2105-15-343

**Published:** 2014-10-04

**Authors:** Mutharasu Gnanavel, Prachi Mehrotra, Ramaswamy Rakshambikai, Juliette Martin, Narayanaswamy Srinivasan, Ramachandra M Bhaskara

**Affiliations:** Molecular Biophysics Unit, Indian Institute of Science, Bangalore, 560012 India; IISc Mathematics Initiative, Indian Institute of Science, Bangalore, 560012 India; Bases Moléculaires et Structurales des Systèmes Infectieux, CNRS, UMR 5086; Université Lyon 1; IBCP, 7 passage du Vercors, F-69367 Lyon Cedex 07, France; National Center for Biological Sciences, Tata Institute of Fundamental Research, GKVK, Bangalore, 560065 India; Molecular Signaling Lab, Signal Processing Department, Tampere University of Technology, Tampere, Finland; Department of Theoretical Biophysics, Max-Planck Institute of Biophysics, Max-von-Laue-Straβe 3, D-60438 Frankfurt am Main, Germany

**Keywords:** Alignment-free comparison, Domain architectures, Multi-domain proteins, Protein classification

## Abstract

**Background:**

The function of a protein can be deciphered with higher accuracy from its structure than from its amino acid sequence. Due to the huge gap in the available protein sequence and structural space, tools that can generate functionally homogeneous clusters using only the sequence information, hold great importance. For this, traditional alignment-based tools work well in most cases and clustering is performed on the basis of sequence similarity. But, in the case of multi-domain proteins, the alignment quality might be poor due to varied lengths of the proteins, domain shuffling or circular permutations. Multi-domain proteins are ubiquitous in nature, hence alignment-free tools, which overcome the shortcomings of alignment-based protein comparison methods, are required. Further, existing tools classify proteins using only domain-level information and hence miss out on the information encoded in the tethered regions or accessory domains. Our method, on the other hand, takes into account the full-length sequence of a protein, consolidating the complete sequence information to understand a given protein better.

**Results:**

Our web-server, CLAP (Classification of Proteins), is one such alignment-free software for automatic classification of protein sequences. It utilizes a pattern-matching algorithm that assigns local matching scores (LMS) to residues that are a part of the matched patterns between two sequences being compared. CLAP works on full-length sequences and does not require prior domain definitions.

Pilot studies undertaken previously on protein kinases and immunoglobulins have shown that CLAP yields clusters, which have high functional and domain architectural similarity. Moreover, parsing at a statistically determined cut-off resulted in clusters that corroborated with the sub-family level classification of that particular domain family.

**Conclusions:**

CLAP is a useful protein-clustering tool, independent of domain assignment, domain order, sequence length and domain diversity. Our method can be used for any set of protein sequences, yielding functionally relevant clusters with high domain architectural homogeneity. The CLAP web server is freely available for academic use at http://nslab.mbu.iisc.ernet.in/clap/.

## Background

With the advent of next generation sequencing methods, there has been an explosion of the sequence information available at our disposal. Therefore, it is increasingly important to consolidate this large amount of sequence data and segregate them into functionally meaningful groups. The current approaches to classify proteins operate at the level of protein domains. Several databases [1, 2] that present classification of protein domains, have contributed tremendously to our understanding of the relationships between protein domain sequences, structures and functions. Majority of the proteins in sequence databases are multi-domain in nature [3]. Multi-domain proteins with similar sequential order of domains tend to have a high functional similarity [4–6]. Domain unassigned regions can also play critical roles in function and/or regulation of proteins. Thus, in addition to the current domain-level based classification, it is important to perform classification of proteins at the entire gene product level.

Traditional alignment-based methods look for homologous regions in the same sequential order in proteins being compared. Hence, they would yield poor alignments in the case of multi-domain proteins, which have undergone domain shuffling or circular permutations [7]. Alignment-free approaches help in overcoming these limitations of alignment-based methods. A wide variety of concepts, like Markov chain models, Kullback–Leibler discrepancy, chaos theory, Kolmogorov complexity, decision tree induction algorithm, graphical representation, and probabilistic measures, have been incorporated in protein sequence comparison methods to calculate evolutionary distances between the sequences [8, 9].

We use an alignment-free algorithm that performs a simple local matching of consecutive five-residue fragment distributions between the two proteins to compute a Local Matching Score (LMS) based on the method developed by Kelil and co-workers [10]. The five-residue length is an optimal choice, which captures the advantages of alignment-based methods (ability to provide residue correspondence) and also overcomes their limitations (speed and inability to align). We have successfully incorporated this algorithm into a pipeline for classification of full-length protein sequences. The superiority of this method over other alignment-based methods has been shown earlier for protein kinases [11] and recently for immunoglobulin domain containing proteins [12]. This method is able to segregate functionally similar proteins, which also share similar domain-architectures [12]. In the case of kinases, CLAP could reproduce clusters corresponding to Hanks and Hunter classification scheme [12]. Through the immunoglobulin data-set we could assess the performance of CLAP in the case of a highly promiscuous and divergent domain family. The results showed that CLAP yielded domain architecturally homogeneous and functionally similar clusters. We believe this method will be effective in the large-scale phylogenomic analysis.

## Implementation

The engine of our classification scheme is the pattern-matching algorithm, which measures local similarities between two protein sequences *s* and *s*′ as LMS (Local Matching Score). It can be depicted as,


Where,  and  are residues from *s* and *s*′ that are a part of all matched five-residue fragments between the two sequences. *M*[*i,i*] is the BLOSUM62 substitution score. The scores computed are normalized to give a distance measure ranging from 0 to 1.


**Figure 1:**
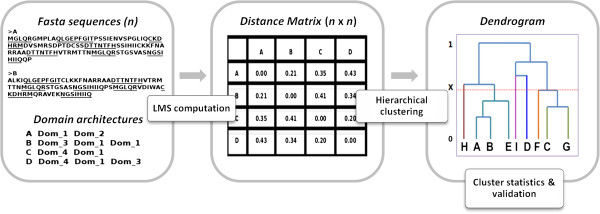
**Schematic of the CLAP server.** Left panel - The inputs to the server are: a set of n protein sequences (Fasta format), a tree parsing cut-off ‘×’, between 0 and 1 (optional) and a tab-delimited file containing domain architecture details for each protein file (optional). Middle panel - A pairwise sequence comparison is performed using the Local Matching Scores method and a normalized distance matrix is computed. Right panel - This distance matrix is subjected to hierarchical clustering using Wards method. The resulting dendrogram is parsed using the user specified cut-off ‘×’. The clusters obtained are analyzed for similarities in domain-architectures.

The pairwise distances computed using LMS are represented as a symmetric square matrix. These distances are subjected to agglomerative clustering based on Ward’s minimum variance method [13] as employed in *hclust* module of R [14]. The hierarchical clustering obtained is represented as a dendrogram that can be parsed at various distance cut-offs (×), ranging from 0 to 1, to obtain distinct clusters. We believe that the clusters generated at a statistically significant cut-off, which maximizes inter-cluster dissimilarity and minimizes intra-cluster dissimilarity, are representative of the subfamily organization in a dataset of protein sequences. The domain architectural similarities and differences of these clusters help in determining sub-family defining features. Figure 1 summarizes the workflow of the web server.

### Server description

The main user interface allows users to input amino acid sequences in Fasta format. The set of sequences can be either pasted into the sequence window or uploaded as a Fasta formatted file. Input data is rigorously checked to ensure a valid input and if any problem is found the appropriate error message is displayed. Unlike other methods, domain annotation is not a pre-requisite for this method. In order to visualize the relationships between the sequences, the distance matrix obtained using LMS based scores is subjected to hierarchical clustering. If the user specifies a cut-off ‘×’ (0 to 1) for parsing the hierarchical tree, clusters are generated and different clusters are shown in separate colors. The coloring is done with the help of A2R library from R statistical package. The coloured dendrogram is available for download in png format.

For a particular cut-off, the cluster index of each sequence is provided in a text file. In case no cut-off has been given, a simple dendrogram is provided in both the EPS as well as Newick formats. An additional feature (optional) of this web server is to compute domain-architectural similarities within each cluster. In order to utilize this feature, the user needs to input a tab-delimited file containing domain architecture details of each protein sequence in the data set. If this option is exercised, a table containing domain-architecture similarity scores for each cluster is output. Three scoring metrics namely, (i) Jaccard index [15] (ii) Goodman-Kruskal γ index [16] and (iii) duplication similarity index [17], capture the three different aspects of domain architectures.

Jaccard index (*J*_*PQ*_) measures the ratio of the number of shared domains to the number of distinct domains between the two proteins being compared. If *N′*_*PQ*_ is the number of shared domains between proteins *P* and *Q,* and *N*_*P*_ and *N*_*Q*_ are the total number of domains belonging to proteins *P* and *Q* respectively, then *J*_*PQ*_ is computed as follows;


Goodman-Kruskal *γ* index (*γ*_*PQ*_) measures the conservation of N-terminal to C-terminal domain order between proteins *P* and *Q*. If *N*^*S*^_*PQ*_ and *N*^*R*^_*PQ*_ are the number of pairs of shared domains in same and in reverse order between proteins *P* and *Q* respectively, then *γ*_*PQ*_ can be calculated as;


*γ*_*PQ*_ score was rescaled to values ranging from 0 to 1.

The duplication similarity [17] index (*D*_*PQ*_) between proteins *P* and *Q* is defined as;


Where,


The means of the above indices (JC-mean, GK-mean and DS-mean) as well as the standard deviations for all combinations of protein pairs within each cluster are provided in a table.

All the result files are provided in a downloadable tar format. This tar file contains information on the LMS-based distance matrix, the dendrogram and the cluster details for each cluster. The web-server also contains Help and FAQ pages.

## Results and discussion

CLAP is a tool for clustering protein sequences that works well with any set of amino acid sequences. The only requirement is the amino acid sequences of the proteins and no information on domain boundaries is required. Another advantage of CLAP is that full-length sequences are taken into account hence utilizing the information contained in accessory domains as well as inter-domain regions.

The assessment of the performance of CLAP, in terms of time efficiency as well as whether it generates biologically meaningful clusters, was reported in our previous paper [12]. In the previous study, two pilot data sets, one of protein kinases and the other of immunoglobulin domain containing proteins were selected from Pfam database [2]. As kinases are well classified, the first data set helped to study if CLAP could reproduce an existing well-established classification. Indeed, the clustering obtained from CLAP showed very little deviation from the well-accepted Hanks and Hunter classification (Table S2; Supplementary file 1 of reference [12]). As for the immunoglobulin data set, CLAP yielded domain-architecturally homogeneous clusters with high functional similarities [12]. Pearsons correlation and t-test, among other statistical tests helped to show that there is no significant correlation between the results from CLAP and ClustalW (an alignment-based method) and that there is a significant difference between the two [12]. However consideration of similarities in functions of proteins and domain architectural similarities within and across clusters clearly showed that the clusters resulted from using CLAP are superior than the clusters suggested by ClustalW [12].

### Execution time of CLAP with respect to other sequence comparison methods

The execution time of CLAP was compared with other existing sequence comparison methods. It has been previously shown that CLAP is ~ 7 times faster than other alignment-based methods that employ dynamic programming [12]. The analysis was repeated for the web-server. Table 1 shows a detailed comparison of the execution time utilized by CLAP, an alignment-free method CLUSS [10], an alignment-based method ClustalW [18], a k-tuple based measure [18] and a sequence identity based clustering method CD-HIT [19]. Though each method has its own limitations, the main aim here however is to compare the execution time of CLAP with other methods for sequence comparison. Alignment-based methods work well if the input sequences correspond to single domain proteins or single domain of multi-domain proteins. With an increase in either the data set size or the length of the protein, the time taken to run these methods increases. Taking small (<100 amino acids), medium (400–600 amino acids) and large (>850 amino acids) single-domain proteins, data-sets containing 500 and 1000 sequences were created. The sequences selected for the analysis are the reviewed entries from Swiss-Prot [20] and hence are well-annotated. As seen from Table 1, the worst that CLAP takes is ~ 10 minutes in the case of a huge dataset of 1000 sequences, whereas the longest that ClustalW takes is ~ 42 minutes for a data set of 500 sequences of large proteins. Although there are cases with CLAP taking more time than alignment-based methods, for most cases, especially for long proteins, CLAP takes shorter time.

**Table 1:** **Clustering of different data-sets of small, medium and large sized protein sequences using different methods**

**Small proteins** (10–100 amino acids length)				
**Number of sequences** - 500				
**Method**	**# of clusters**	**Threshold**	**Word-length**	**Time**
CW	15	0.5	NA	0 m 11.835 s
k-tuple	3	0.5	2	0 m 1.539 s
CLAP	7	0.5	5	2 m 28.322 s
CLUSS	68	NA	4	0 m 11.000 s
CD-HIT	223	0.5	3	0 m 0.034 s
**Small proteins** (10–100 amino acids length)				
**Number of sequences** - 1000				
**Method**	**# of clusters**	**Threshold**	**Word-length**	**Time**
CW	23	0.5	NA	0 m 59.788 s
k-tuple	3	0.5	2	0 m 5.659 s
CLAP	17	0.5	5	9 m 52.099 s
CLUSS	NA	NA	NA	0 m 11.000 s
CD-HIT	607	0.5	3	0 m 0.091 s
**Medium proteins** (400–600 amino acids length)				
**Number of sequences** - 500				
**Method**	**# of clusters**	**Threshold**	**Word-length**	**Time**
CW	2	0.5	NA	8 m 46.895 s
k-tuple	3	0.5	2	0 m 2.25 s
CLAP	3	0.5	5	2 m 50.918 s
CLUSS	95	NA	4	0 m 3.133 s
CD-HIT	227	0.5	3	0 m 0.592 s
**Medium proteins** (400–600 amino acids length)				
**Number of sequences** - 1000				
**Method**	**# of clusters**	**Threshold**	**Word-length**	**Time**
CW	5	0.5	NA	32 m 50.379 s
k-tuple	2	0.5	2	0 m 7.789 s
CLAP	7	0.5	5	11 m1 2.664 s
CLUSS	NA	NA	NA	NA
CD-HIT	708	0.5	3	0 m 3.281 s
**Large proteins** (850–1000 amino acids length)				
**Number of sequences** - 500				
**Method**	**# of clusters**	**Threshold**	**Word-length**	**Time**
CW	15	0.5	NA	42 m 1.184 s
k-tuple	4	0.5	2	0 m 2.91 s
CLAP	4	0.5	5	4 m 22.752 s
CLUSS	NA	NA	NA	NA
CD-HIT	125	0.5	3	0 m0.916 s

The processing time was computed using the workstation that hosts the CLAP web-server, with a 2.40 GHz, Intel xeon processor and 16GB RAM running CentOS. The number of clusters generated at a specific threshold and word-length used in the computations is also shown.

### Comparison of clustering obtained from CLAP and ClustalW for varying lengths of proteins

**Figure 2:**
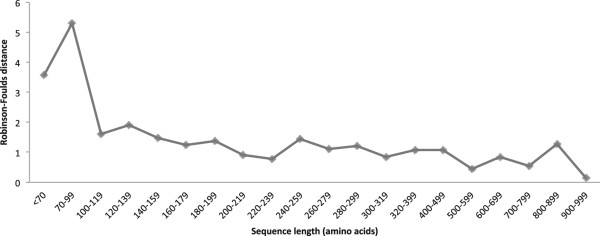
**Plot of Robinson-Foulds (RF) distance between dendrograms from CLAP and ClustalW with respect to different sizes of proteins sequences.**

To quantify the differences in the results of CLAP and ClustalW, we used Robinson-Foulds distance (RF) as implemented in the Hash-RF algorithm [21]. This distance metric measures the difference in branching patterns of two unrooted trees. Data sets of lengths ranging from 10–70, 70–99, 100–119, 120-139…300-319, 320–399, 400-499…900-999 were constructed. Sequences of single domain proteins have been extracted from Swiss-Prot [20]. Taking only single-domain proteins ensured that the performance of ClustalW will be unaffected by phenomena like domain shuffling and circular permutations. The RF distances of the different data sets have been plotted as shown in Figure 2. For small proteins of size < 100 amino acids, CLAP and ClustalW dendrograms differ significantly (RF distance = 5.292), but both are more or less similar (RF distance is between 0.154 and 1.912) for data sets with proteins longer than 100 amino acids.

### Presentation of results by CLAP server

The output files provided to the user are: a distance matrix (containing all pairwise distances computed using LMS scores), a dendrogram (to visualize the hierarchical clustering), clusters details of each sequence (if cut-off is input by user), and within cluster domain architectural similarities. A tar file containing all the output files is also available for download.

In future, a statistically determined tree cut-off would be suggested to the user, which will generate a clustering that corresponds to an optimal classification of given proteins. An additional feature to be included later is computing functional similarities for each cluster. This would help in understanding the functional similarities and differences among the clusters, eventually leading towards defining sub-families for a specific protein family. In the long run, given an uncharacterized protein sequence, we can accurately predict which sub-family it belongs to, thus achieving its detailed functional classification.

## Conclusions

The sequence space is increasing at a more rapid pace than the rate at which experimental characterizations for these newly found sequences can be performed. Studying the key similarities and differences among a set of proteins will help in defining a sub-family level classification. Hence, tools that accurately predict the functional relationships among protein sequences at family as well as sub-family level are of immense importance. Traditional methods that achieve this aim are alignment-based and utilize only domain level information. This approach may not yield proper results in the case of multi-domain proteins.

Our tool, CLAP uses an alignment-free approach towards protein classification of any given set of sequences. In earlier studies we have shown that CLAP is faster and better than other alignment-based tools. It efficiently clusters protein sequences into functionally meaningful groups having high domain architectural similarities. At a statistically determined cut-off the resulting clustering corresponds to sub-family level classification. Thus, CLAP with an easy to use interface provides a huge step towards efficient protein classification, especially that of multi-domain proteins.

## Availability and requirements

**Project name:** CLAP server

**Project home page:**http://nslab.mbu.iisc.ernet.in/clap

**Operating system(s):** Windows, Linux, Mac

**Programming language:** C++, R, Perl

**Any restrictions to use by non-academics:** license needed

## Authors’ information

Mutharasu Gnanavel and Prachi Mehrotra are joint first authors.

## Abbreviations

CLAP: Classification of proteins
LMS: Local Matching Score.
